# Unusual spermine-conjugated hydroxycinnamic acids on pollen: function and evolutionary advantage

**DOI:** 10.1093/jxb/ery359

**Published:** 2018-11-26

**Authors:** Thomas Vogt

**Affiliations:** Leibniz Institute of Plant Biochemistry, Department of Cell and Metabolic Biology, Halle (Saale), Germany

**Keywords:** Acylated spermine, BAHD acyltransferases, *Cichorium intybus*, metabolic diversification, pollen coat, phenolamides, SHTs

## Abstract

This article comments on:

**Marianne Delporte, Guillaume Bernard, Guillaume Legrand, Björn Hielscher, Arnaud Lanoue, Roland Molinié, Caroline Rambaud, David Mathiron, Sébastien Besseau, Nicole Linka, Jean-Louis Hilbert, and David Gagneul**. 2018. A BAHD neofunctionalization promotes tetrahydroxycinnamoyl spermine accumulation in pollen coat of the Asteraceae family. Journal of Experimental Botany **69,** 5355–5371.

**Conjugates between polyamines and hydroxycinnamic acids are found on the pollen surface of all higher plants, both mono- and dicots. But we don’t know why they are there. Delporte *et al.* (2018) have now shown that in the tapetum of the Asteraceae (sunflower family) a new type of BAHD-acyltransferase is expressed, able to transfer coenzyme A-activated coumaric acid to all four primary and secondary amine groups of the polyamine spermine. In the case of chicory this sequential addition results in a fully substituted tetracoumaroyl–spermine conjugate and points to an evolutionary advantage of these functionally enigmatic compounds.**

This story begins 40 years ago, when large amounts of unusual conjugates were detected in the male reproductive organs of maize (Martin-Tanguy *et al.*, 1978). These were hydroxycinnamic acids (HCAs), like ferulic acid, linked to the individual amino groups of putrescine and spermidine. Putrescine and its biosynthetic descendants spermidine and spermine, which are all polyamines, are derived from ornithine and arginine by decarboxylation and sequential aminopropyltransfer from decarboxylated adenosylmethionine via spermidine and spermine synthase, respectively (Martin-Tanguy, 2001). The high content of these HCA–amide (HCAA) conjugates in maize pollen was initially linked to plant fecundity, until it was shown that they are present throughout the plant kingdom. Their high concentration specifically in pollen grains remains a mystery.

Further characterization followed in the 1980s with the rise of chromatographic and analytical tools and an increased interest in chemotaxonomy. Several studies using mass spectrometry and NMR of a variety of spermidine-conjugated HCAAs from pollen of wind-pollinated species like birch and hazel showed localization on the surface of pollen grains and a more precise structural identification (Meurer *et al.*, 1988). Initially, only mono- and bis-substituted conjugates were identified which were later shown to be present in flowers, leaves, seeds or roots of all plant species investigated (Luo *et al.*, 2009). The first fully substituted HCAA, *N*^1^, *N*^5^, *N*^10^-tricoumaroyl spermidine, was isolated and described from the pollen of *Crataegus* (hawthorn) and was also detected in several other Rosaceae (Strack *et al.*, 1990). Initial speculation that these compounds could be used as a chemotaxonomic marker failed, but it turned out that tris-substituted conjugates were indeed pollen specific and apparently exclusively synthesized in the tapetum of developing flowers through the action of a new type of a BAHD-type acyltransferase, spermidine hydroxycinnamoyltransferase (SHT) (Grienenberger *et al.*, 2009).

Although not of chemotaxonomic significance, in a comprehensive study Elejalde-Palmett (2015) showed that the products of SHT, tris-substituted conjugates, can be regarded as a marker of the pollen exine of eudicotyledons: they are always present. Neither these compounds nor any corresponding SHT-like sequences have so far been reported from monocots or gymnosperms. While the product profile of SHTs of Rosaceae appeared restricted to uniform coumaric acid derivatives of spermidine, Arabidopsis SHT is co-expressed with tapetum-specific CYP98A8 and an *O*-methyltransferase, AtTSM1 (Ehlting *et al.*, 2008; Fellenberg *et al.*, 2008; Matsuno *et al.*, 2009), resulting in downstream hydroxylations and a single methylation towards a diverse pattern of mono-, bis- and tris-substituted HCAAs which is characteristic for the Brassicaceae (Matsuno *et al.*, 2009; Handrick *et al.*, 2010).

Two additional BAHD-like acyltransferases, denoted SCT and SDT, are expressed in the Arabidopsis tapetum, but only SHT acts to synthesize the major tris-feruloyl and sinapoyl-based spermidine conjugates (Grienenberger *et al.*, 2009). Similar to the biosynthesis of sporopollenin polymers of the exine, phenolamide metabolism is probably associated in a metabolon and links polyamine to phenylpropanoid metabolism (Bassard *et al.*, 2010; Lallemand *et al.*, 2013). Although spermine is also present in Arabidopsis anthers in high concentrations Arabidopsis SHT is selective for spermidine. SHT knockout mutants, which are virtually devoid of HCAAs, showed no effect on the level of free polyamines and do not result in an increase of pollen-specific flavonol sophorosides (Fellenberg *et al.*, 2012; Yonekura-Sakakibara *et al.*, 2014) pointing to different pools of precursors and independent pathway organization for both types of phenylpropanoids in the tapetum.

## New BAHD-type enzyme in the tapetum of Asteraceae

Asteraceae (sunflower family) were the focus of the work by Delporte *et al.* (2018). It is the most-rich angiosperm family, with rapid diversification over the last 60 million years specifically in open habitats all over the world (Barreda *et al.*, 2015). Besides sunflower, many other Asteraceae have been used for nutrition (e.g. artichoke, *Cynara scolymus*), as herbal medicines (*Arnica* and *Echinacea* spp.) or, most recently, as an alternative source of rubber (dandelion, *Taraxacum offininale*). Roasted chicory (*Cichorium intybus*) rootstocks were used extensively in the last century as a cheap coffee replacement. Due to its high content of inulin, a 1,3-linked carbohydrate, it is currently considered as a source of dietary fiber and promoted as ‘functional food’ (Stolze *et al.*, 2017).

Asteraceae are usually pollinated by insects and attractive floral organs have contributed to their worldwide success and promoted the evolution of insects, such as solitary bees (Barreda *et al.*, 2015). Delporte *et al.* (2018) identified two new members of the SHT clade of BAHD-acyltransferases in Asteraceae, CiSHT1 and CiSHT2, and extend the biosynthetic diversity of HCAAs in reproductive organs of plants (Delporte *et al.*, 2018). LC-MS-based analysis identified a tetra-substituted coumaroyl-CoA spermine as the major phenolamide in methanolic pollen washes of chicory besides minor spermidine conjugates which can be bis- or tris-substituted (Box 1).

**Box 1. Cladogram of individual clusters of enzymes required for differentially substituted HCAA**

The cladogram shows the difference between the new tetracoumaroyl spermine conjugates (1) synthesized via CiSHT1 and CiSHT2 compared to the previously identified clusters of tris (2) and bis (3)-substituted spermidine derivatives. Functionally characterized SHTs and SDT are marked in red. Based on sequence identities of the SHTs, the set of tetra-substituted compounds appears to be restricted to the Asteraceae, but further analysis of metabolites and functional characterization of the corresponding enzymes is required.

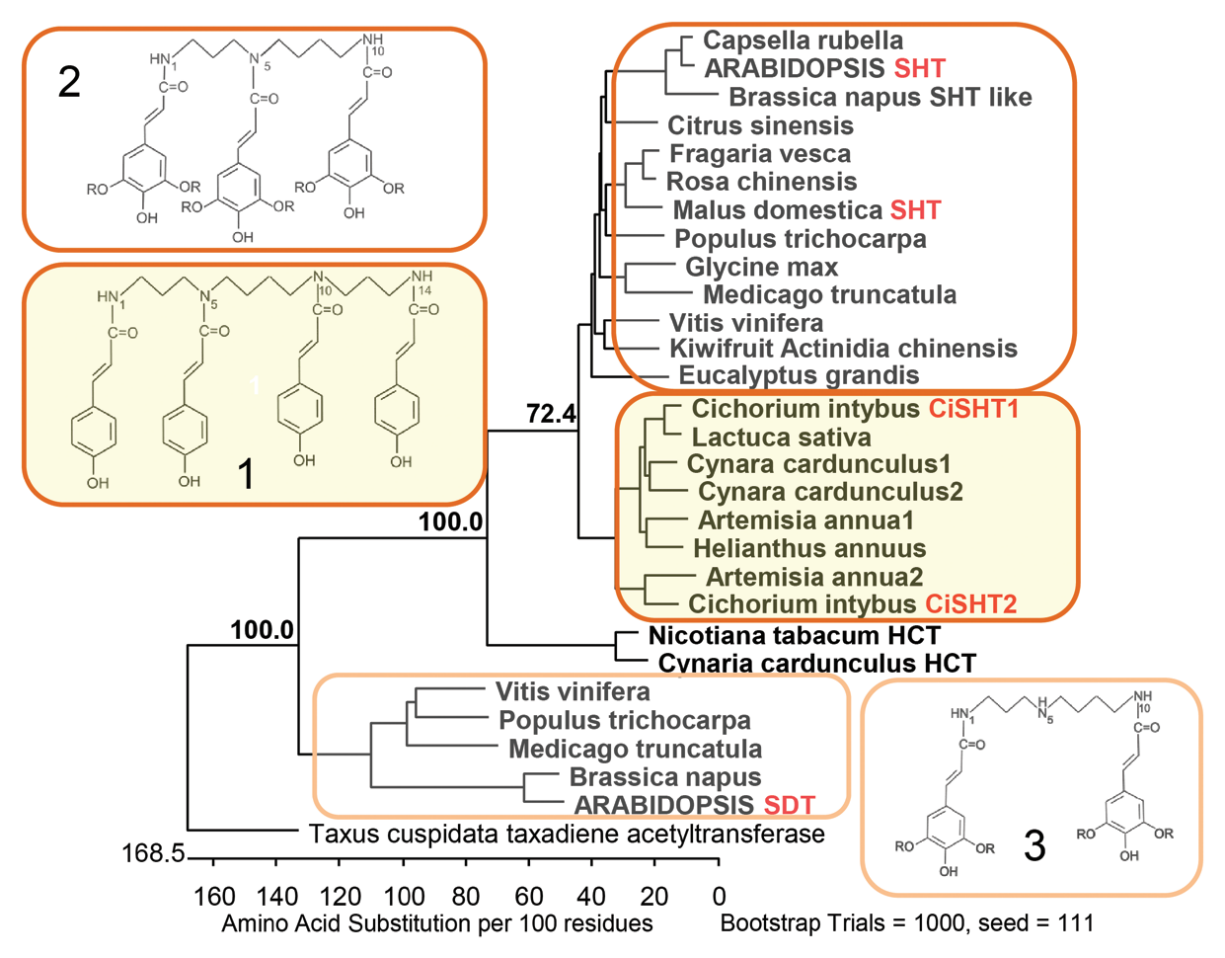

At first glance this extended acceptor specificity may not seem spectacular, but requires multiple changes in terms of substrate availability, transport, regulation and (last but not least) changes in the amino acid sequence of the two chicory CiSHTs identified by Delporte *et al.* (2018). New enzyme functions are based on divergent evolution, gene duplication and subsequent random functional re-differentiation of established features (Lynch and Katju, 2004; Aharoni *et al.*, 2005). A closer inspection of the active site and site-directed mutagenesis of this enzyme might reveal that only a single amino acid change leading to a somewhat larger hydrophobic cavity, capable of hosting bulky acylated spermine derivatives, could result in this minor shift of substrate preference. Recombinant BAHDs are usually difficult to express in microbial systems and the currently solved BAHD-crystal structures belong to only distantly related BAHD subclades compared to CiSHTs (Unno *et al.*, 2007). In functionally related serine carboxypeptidase-like enzymes (SCPLs) a simple Glu to Asp change in a catalytic triade resulted in a surprisingly complete functional change from a peptide hydrolase to a 1-*O*-glucose ester transferase. Instead of water as in the case of a hydrolase, the sinapoyl residue is now transferred to malic acid resulting in sinapoylmalate, a typical metabolite in Brassicaceae leaves (Stehle *et al.*, 2009). Nature seems to favour simple and elegant solutions.

Crystallization of the enzyme and a close-up view of the active site by modelling and docking studies of CiSHT should shed light on the new specificity and might also reveal the differences in CiSHT1 and CiSHT2 in terms of functional expression. All other features of the chicory BAHDs appear conserved compared to other BAHDs, including high activity at alkaline pH, promiscuous substrate CoA-donor and amine-acceptor preference *in vitro* and *in vivo*, even when expressed ectopically in Arabidopsis, and localization in the cytoplasm, as demonstrated by YFP-fusions. A tight association of SHT in a complex appears plausible, since only the fully substituted HCAA is detected in large quantities *in vivo* and no isomerization of the product is observed.

Compared to the complex substitution pattern of Arabidopis, the simple tetra-coumaroyl spermine pattern in chicory already points to evolutionarily independent diversification in the Brassicaceae and Asteraceae. Additional downstream modifications, like hydroxylation and subsequent methylation, appear to be missing in chicory and presumably are less relevant for their biological function. The modified acceptor specificity, of course, now requires verification in other tribes of the Cichorioideae and Asteroideae, since the reported sequence identities are insufficient to draw precise conclusions on acceptor preference. It appears plausible that gene duplication and neo-functionalization in the Asteraceae occurred before the divergence of the eudicots, but after the tapetal SHT and the accumulation of tris-substituted HCAAs were established. Now, with emerging sequencing of complete genomes of ‘primitive’ dicots like *Amborella* a more precise annotation of SHT divergence and evolution might be possible (The Amborella Genome Project, 2013).

## Enigmatic roles of SHT and resulting metabolites: many questions

The universal deposition of HCAAs late in pollen development is usually regarded as a final decoration step in the highly coordinated assembly of the male gametophyte (Ariizumi and Toriyama, 2011). After mechanistic and regulatory steps in this process have been solved, the most intriguing question remains unanswered: what is the function of these compounds on the pollen surface and what exactly is the advantage to pollen fitness? Also, why is there conserved expression of SHT-type BAHDs specifically in the male reproductive organs of all higher dicots? And finally, is there any advantage of the shift from spermidine- to spermine-conjugated HCAs? To quote the famous biochemist Erwin Chargaff (1905–2002): ‘science is wonderfully equipped to answer the question “how?” but it gets terribly confused when you ask the question “why?”’ Are we confused or is it just the lack of suitable tools or the complexity of the problem which impedes identification of one or several functions?

Mo et al. (1992) unexpectedly restored the fertility of chalcone synthase deficient and conditionally sterile petunia plants (Napoli *et al.*, 1990) by including micromolar concentrations of flavonols to the germination medium. Therefore, the flavonol diglycosides which, besides HCAAs, accumulate on the exine of higher dicots, could at least be associated with pollen fertility in these two species. However, in all other species tested so far, including Arabidopsis, this requirement could not be verified. How exactly these flavonols contribute to pollen germination remains enigmatic even today. Fertility in SHT and HCAA-deficient Arabidopsis plants appears unimpaired, although cracks in a small percentage of *sht* pollen grains could indicate a minor structural role of HCAAs in the pollen wall (Grienenberger *et al.*, 2009). Based on the current data, a general requirement of phenylpropanoids for fecundity of eudicot pollen is not evident, although a supportive role in the numerous factors regulating pollen germination and pollen tube guidance cannot be ruled out (Hafid *et al.*, 2016).

A universal and most obvious function of phenylpropanoids in general is UV protection (Youhnovski *et al.*, 2001). HCAAs, either spermidine- or spermine-linked, show absorbance maxima of 315–330 nm, flavonol glycosides absorb at 354 nm, and a combination of both covers the UV-B and UV-A spectrum of sunlight perfectly. Protection of the genetic information of the pollen grains from damaging UV radiation is consistent with an established role of phenylpropanoids in UV protection (Jansen *et al.*, 1998). Decoration of all four nitrogen atoms of spermine with HCAs increases the UV absorbance of a single molecule by roughly 30% compared to spermidine (which only contains three nitrogen atoms) without affecting the concentration of the metabolites, since the extinction coefficients of individual phenylpropanoids add up. If transport to the pollen surface by an ABC-transporter can be demonstrated for hydropobic HCAAs as postulated for polyketide-derived exine polymers (Quilichini *et al.*, 2014; Lefèvre and Boutry, 2018) then the same number of transported molecules, in an ATP-dependent process, would result in a more efficient way to generate a pollen surface of increased UV absorbance, independent of the decorations of individual phenylpropanoids, which all show similar extinction coefficients. The identification of SHTs in the Asteraceae by sequence identities in this report requires support by identification of the corresponding HCAA-pattern and analysis of SHT-product specificity in other Asteraceae.

In addition to a very plausible role of HCAAs in UV protection, and the usual claims of antioxidant and free radical scavenger potential, a biological role in plant–microbe and plant–pollinator interaction, although difficult to prove, deserves more attention. Although antimicrobial activities of phenylpropanoids and specifically HCAAs have been reported (Daglia, 2012; Tanabe *et al.*, 2015), a specific advantage of the new HCAA pattern in Asteraceae pollen compared to pollen of other species is not immediately obvious. In contrast, a much more fascinating and largely unexplored area is the potentially beneficial effect of these types of compounds in plant–pollinator interactions. The flavonol content and resulting high UV absorbance in flowers of various *Petunia* spp. were already linked to pollinator preference (Sheehan *et al.*, 2016). Therefore, dark spots in flowers could be attractive to some insects, such as bees, which can perceive UV light (Briscoe and Chittka, 2001; Barreda *et al.*, 2015). Most intriguing, tris-coumaroyl spermidine, besides lipids and flavonols from sunflower pollen (Asteraceae), was reported as an insect feeding stimulant (Lin and Mullin, 1999). One should remember that a spermine molecule contains four nitrogen atoms which might be reused by insects upon cleavage of the amide bond. If harvest and distribution of pollen grains could be enhanced by an optimized blend of ‘tasty’ metabolites, this could at least in part account for the rapid success and evolutionary advantage of angiosperms (and specifically the Asteraceae) in their coevolution with insects, potentially accelerating the adaptive radiation of this family of angiosperms and leading to their current diversity. The biological story may be more complex. A clear proof of these assumptions requires precise analytics, mutant analyses, pollen viability studies under enhanced UV light, and insect feeding studies, preferentially under natural, non-greenhouse light conditions.
